# Bempedoic Acid: A New Tool for LDL-Cholesterol Control in Patients with Coronary Artery Disease

**DOI:** 10.31083/j.rcm2305156

**Published:** 2022-04-26

**Authors:** Claudio Bilato, Giorgio Sesti, Maurizio Averna

**Affiliations:** ^1^Division of Cardiology, West Vicenza General Hospitals, 36071 Arzignano-Vicenza, Italy; ^2^Department of Clinical and Molecular Medicine, University La Sapienza, 00185 Rome, Italy; ^3^Department of Health Promotion Sciences Maternal and Infantile Care, Internal Medicine and Medical Specialties, University of Palermo, 90127 Palermo, Italy

**Keywords:** bempedoic acid, ATP citrate lyase, LDL-cholesterol, lipid-lowering treatment, novel LDL-C treatment

## Abstract

Nowdays a small proportion of patients with high/very high/extreme atherosclerotic cardiovascular disease risk achieves the optimal target of LDL-cholesterol, because of drug intolerance, poor adherence to the therapy, or inapplicability of the stepwise strategy in lipid lowering therapy, recommended by the current guidelines. The new oral agent bempedoic acid lowers plasma LDL-cholesterol by inhibiting adenosine triphosphate-citrate lyase, an enzyme involved in the synthesis of cholesterol, and, ultimately, by up-regulating the LDL receptors. Several clinical trials in patients with atherosclerotic cardiovascular disease or familial heterozygous hypercholesterolemia demonstrated that bempedoic acid alone or combined with statins and/or ezetimibe significantly reduced LDL-cholesterol and high-sensitivity C-reactive protein. Bempedoic acid is well tolerated with no significant increase in muscle-related symptoms, since it can be activated only in the liver but not in the skeletal muscles. Bempedoic acid provides an effective tool to further reduce LDL-cholesterol as add on therapy in patients unable to reach the target despite maximally tolerated lipid lowering therapy.

## 1. Introduction

According to the current European Society of Cardiology/European Atherosclerosis Society guidelines on managment of dyslipidemia, LDL-cholesterol (LDL-C) below 55 mg/dL and a 50% reduction of the baseline LDL-C value are recommended in patients with very high cardiovascular (CV) risk [1]. Indeed, the currently available drug treatments are able to reduce LDL-C up to 85% of the basal levels. However, despite the efficacy of the lipid lowering therapy, a very small proportion of coronary artery disease (CAD) patients reaches the recommended lipid targets. In the DA VINCI study [2] which enrolled 2888 secondary prevention patients from 18 European countries, the target of LDL-C ≤55 mg/dL was achieved only in the 18% of the population. Similarly, among 10,071 patients with previous percutaneous coronary intervention, only 23% showed a LDL-C value below 55 mg/dL. Moreover, fewer than 6% of the subjects with recurrent vascular events achieved the LDL-C target of <40 mg/dL, which is the recommended threshold for these extremely high CV risk individuals [3]. Several reasons explain these disappointing results, such as drug intolerance, poor adherence to the therapy, or the lack of “applicability” of the recommended stepwise strategy in lipid lowering therapy. Whatsoever the reason, new lipid lowering treatments are needed in order to reach the recommended LDL-C target in a significantly higher proportion of patients with CAD. Among these, bempedoic acid (BA) will play an important role in achieving the more stringent LDL-C goals in the very high risk subjects, by complementing the current treatments and facilitating personalized therapy.

## 2. Bempedoic Acid is a First-in-Class Inhibitor of Adenosine Triphosphate-Citrate Lyase

BA is a small molecule, which can be administered orally, as prodrug, once daily as a single dose of 180 mg. It is rapidly absorbed in the small intestine and has a half-life of 15–24 hours. BA oral bioavailability is not affected by food, nor its pharmacokinetic properties by age, sex, race, or weight. BA inhibits adenosine triphosphate-citrate lyase (ACL). ACL is an enzyme involved in the cholesterol synthesis pathway, acting upstream of the hydroxy-methylglutaryl coenzyme A reductase (HMGCR). Results from Mendelian randomization studies suggest that inhibiting ACL lowers plasma LDL-C levels in the same way that inhibiting HMGCR by a statin does—that is, through up-regulation of the LDL receptors. Moreover, genetic variants that mimic the effect of ACL inhibitors lower the risk of CV events [4]. Furthermore, BA lowers plasma glucose by activating adenosine $5^{\prime }$-monophosphate-activated protein kinase, which inhibits gluconeogenesis and suppresses the hepatic production of glucose [5].

As prodrug, BA needs to be converted to its active form, bempedoyl-CoA, by the enzyme very-long-chain acyl-CoA synthetase-1 (ACSVL1). Because ACSVL1 is expressed in the hepatocytes but not in adipose tissue, intestine, or skeletal muscle [6], BA should cause considerably less muscle-related adverse effects compared to statin therapy [7]. The fact that bempedoyl-CoA is not found in the plasma of individuals treated with bempedoic acid and likely it does not escape the hepatocyte suggests that its activity is limited to the liver and may contribute to the reduction of the muscle-related adverse effects [8].

The glucuronides of BA and bempedoyl-CoA are the major metabolites found in the plasma. BA is eliminated mainly by the kidneys, with 70% recovered in urine and 30% in feces [8].

*In vitro* metabolic interaction studies suggest that BA is not metabolized by and does not inhibit or induce cytochrome P450 enzymes. As a result, drug-drug interactions with drugs metabolized by this path, including warfarin, are not anticipated. BA inhibits organic anion transporter OAT2 *in vitro*, which plays a role in uric acid and creatinine uptake from blood to proximal tubular cells, and may explain the minor elevations in serum creatinine and uric acid [5]. The most significant drug-drug interactions involve BA and simvastatin and pravastatin. Co-administration of simvastatin 40 mg with BA 180 mg in healthy participants, for example, caused an approximately 2-fold and 1.5-fold increase in simvastatin AUC and Cmax, respectively. Considering that many patients under BA treatment concomitantly take statins, a certain grade of caution should be kept and doses of simvastatin >20 mg (and pravastatin >40 mg) should be avoided [8].

Based on the above characteristics, BA represents a novel therapeutical option to effectively reduce LDL-C, as demonstrated by the CLEAR program, which encompasses four clinical trials on the safety and the efficacy of BA in a wide range of patients.

## 3. Clinical Evidences

So far, five phase III randomized clinical trials have been published (Table 1, Ref. [9, 10, 11, 12, 13]). The CLEAR program includes four studies. The CLEAR Harmony [9] and the CLEAR Wisdom [10] trials enrolled 3009 individuals with previous atherosclerotic cardiovascular disease (ASCVD) and/or heterozygous familial hypercholesterolemia (HeFH) and elevated LDL-C despite the treatment with maximally tolerated statin therapy. The follow-up period was 52 weeks but the Harmony trial will also provide long-term safety results in its open-label extension (OLE) up to an extra 82 weeks of follow-up [14]. By contrast, the CLEAR Tranquility [11] and the CLEAR Serenity [12] trials enrolled 614 patients with hyperlipidemia and statin intolerance. In these studies the follow-up was 24 weeks maximum. All the studies were conducted between 2016 and 2018 at European, US and Canadian sites. The primary end point of the CLEAR Harmony trial was the overall safety, based on the occurrence of adverse events and/or clinical safety laboratory findings. The average percent reduction in LDL-C at week 12 was the major secondary end point in the CLEAR Harmony study and the primary end point in the CLEAR Wisdom, Tranquillity and Serenity trials. In all studies, additional secondary/tertiary end points included percent reduction of LDL-C at week 24 and/or absolute or percentage changes in total cholesterol, non-HDL-C, apolipoprotein B, and high-sensitivity C-reactive protein at different time points. In general, adverse events of particular attention embraced hepatic and renal events, muscle-related symptoms, hyperuricemia, gout, metabolic acidosis, hypoglycemia or new-onset or worsening diabetes, and neurocognitive disorders. Fasting triglycerides ≥500 mg/dL, glomerular filtration rate <30 mL/min per 1.73 $m^{2}$, nephropathy, body mass index ≥50 kg/$m^{2}$, uncontrolled hypertension, uncontrolled hypothyroidism, liver disease, conditions affecting drug absorption, hematologic or coagulation disorders, active malignancy, or creatine kinase elevations >3 ULN were among the exclusion criteria. Baseline LDL-C levels across these four trials ranged from 103.2 to 157.6 mg/dL. As expected, BA reduces LDL-C depending on the lipid-lowering background therapy. A recent pooled analysis of four RCTs showed that among patients with ASCVD and/or HeFH receiving a maximally tolerated statin, the LDL-C level percentage change, from baseline to week 12, was –17.8% (placebo corrected, 95% CI, –19.5% to –16.0%; *p *< 0.001). On the other hand, among patients with statin intolerance, the percentage reduction in LDL-C levels at week 12 was –24.5% (placebo corrected, 95% CI, –27.8% to –21.1%; *p *< 0.001). The reduction in LDL-C levels with BA was sustained during long-term follow-up in both groups of patients [5]. In addition to LDL-C lowering, BA improved other parameters such as total cholesterol, non-HDL-C, apolipoprotein B, and high sensitive C-reactive protein (hs-CRP) consistently across the different trials, as reported in Table 2.

**Table 1.** **Currently available Phase III randomized clinical trials**.

Study	Duration	Population	Treatment groups (n)	LDL-C reduction at 12 weeks (BA versus placebo)	Muscle symptoms (BA versus placebo)
CLEAR Harmony [9]	52 weeks	ASCVD and/or HeFH patients with LDL-C ≥70 mg/dL in maximally tolerated statin with or without other LLT	BA (n = 1488)	–18.1% 95% CI, –20.0, –16.1	13.1% vs 10.1%
			placebo (n = 742)	*p *< 0.001	
CLEAR Wisdom [10]	52 weeks		BA (n = 522)	–17.4% 95% CI, –21.0, –13.9	Not available
			placebo (n = 257)	*p *< 0.001	
CLEAR Tranquility [11]	12 weeks	Hypercholesterolemic patients with statin intolerance requiring additional LDL-C lowering	BA (n = 181)	–28.5% 95% CI, –34.4, –22.5	1.7% vs 2.3%
			placebo (n = 88)	*p *< 0.001	
CLEAR Serenity [12]	24 weeks		BA (n = 234)	–21.4% 95% CI, –25.1, –17.7	12.8% vs 16.2%
			placebo (n = 111)	*p *< 0.001	
Ballantyne [13]	12 weeks	ASCVD and/or HeFH or multiple CVD risk factors	BA (n = 110)	BA: –19.0%	BA: 8.0%
			Ezetimibe (n = 109)	Ezetimibe: –25.0%	Ezetimibe: 8.1%
			BA + Ezetimibe (n =108)	BA + Ezetimibe: –38.0% 95% CI, –46.5, –29.6	BA + Ezetimibe: 7.1%
			Placebo (n = 55)	*p *< 0.001	Placebo: 7.3%

Abbreviations: ASCVD, atherosclerotic cardiovascular disease; BA, Bempedoic Acid; CI, confidence interval; HeFH, heterozygous familial hypercholesterolemia; LDL-C, low-density lipoprotein cholesterol; and LLT, lipid lowering therapy.

**Table 2.** **BA-mediated lipid profile modifications from baseline to week 12 in the CLEAR Program**.

	ASCVD/HeFH on statin			statin intolerant		
	% reduction (placebo-corrected)	95% CI	*p*	% reduction (placebo-corrected)	95% CI	*p*
total cholesterol	–11.1	–12.2, –9.9	<0.001	–16.2	–18.4, –13.9	<0.001
LDL cholesterol	–17.8	–19.5, –16.0	<0.001	–24.5	–27.8, –21.1	<0.001
non-HDL cholesterol	–13.1	–14.7, –11.6	<0.001	–20.4	–20.4, –17.5	<0.001
apolipoprotein B	–12.1	–13.6, –10.7	<0.001	–16.9	–19.6, –14.2	<0.001
hs-CRP	–18.1	–22.7, –13.5	<0.001	–27.4	–36.1, –18.5	<0.001

ASCVD indicates atherosclerotic cardiovascular disease; HeFH, heterozygous familial hypercholesterolemia.

As said before, the Clear Harmony trial was extended in an open-label follow-up to 82 weeks. The definitive results are not yet available in the literature. However, some preliminary observations [14] have shown that BA therapy guaranteed a enduring LDL-C lowering in the long period with an elevate (86.2%) patient adherence. Moreover, during the open-label follow-up no different safety issues emerged compared to the original Harmony study and the overall BA phase 3 CLEAR clinical program.

The efficacy of BA in association with ezetimibe has been studied in a fixed-dose combination (FDC) trial, which included patients at high CVD risk because of the presence of ASCVD, HeFH or multiple CVD risk factors. In this study, the combination of BA plus ezetimibe reduced LDL-C by –38.0% at 12 weeks (placebo-corrected, *p *< 0.001). 67.5% and 31.3% of the patients treated with FDC for 12 weeks achieved LDL-C below 100 mg/dL and 70 mg/dL, respectively, compared to 17.5% and 0% for placebo (*p *< 0.001), 42.5% and 10.0% for ezetimibe (*p *< 0.002) and 43.9% and 6.1% for BA (*p *< 0.003). The effect on LDL-C was similar across all the subgroups, including patients receiving high-intensity (20–40 mg/day of rosuvastatin; 40–80 mg/day of atorvastatin), other-intensity (all the other statins and doses), or no statin therapy. The known differences in the mechanisms of action of BA and ezetimibe were responsible of the additive effect observed in FDC treatment group. Furthermore, it is noteworthy that: (1) 33.7% of the FDC individuals showed >50% reduction in LDL-C baseline levels and (2) at 12 weeks hs-CRP decreased by 35.1% with the BA and ezetimibe FDC compared to 21.6% increase observed in the placebo arm [13].

BA is effective also with other lipid lowering agents, such as proprotein convertase subtilisin/kexin type 9 inhibitors (PCSK9i): in a small phase 2, randomized, double-blind, placebo-controlled study on 57 patients, BA added to background PCSK9i therapy significantly lowered LDL-C by 30.3% (*p *< 0.001) versus placebo, as well as apolipoprotein B, non–HDL-C, total cholesterol (*p *< 0.001 for all), and hs-CRP (*p* = 0.029) [15].

RCTs have undoubtedly shown that lowering LDL-C with statins reduces cardiovascular events in both the primary and the secondary prevention setting with a linear relationship between degree of LDL-C lowering and clinical benefit [16]. Preliminary data from phase III trials on BA suggest a reduction of cardiovascular events according to the achieved LDL-C reduction: so far, the pooled data from these phase III trials are encouraging with a risk reduction of composite cardiovascular outcome (cardiovascular death, nonfatal myocardial infarction, nonfatal stroke, hospitalization for unstable angina, and coronary revascularization) of 25% (RR 0.75, 95% CI 0.56 to 0.99) [17]. However, definite insights will come from the CLEAR Outcomes trial that will determine whether BA added to standard medical therapy reduces the incidence of major cardiovascular events in high risk patients with statin intolerance during an expected median duration of 3.5 years [18].

As far as safety is concerned, there is evidence from pooled analysis, encompassing 3623 patients, that the most common treatment-emergent adverse events (TEAE) did not differ between treatment groups. Other TEAEs of special interest were quantitatively low with a <2% difference in frequency between groups. Compared to placebo, BA treatment is associated with a slight increase in serum uric acid levels (0.5% and 2.1%, *p* = 0.001) and a greater incidence of gout (0.4 versus 1.4%, *p* = 0.008) [7]. The mean difference in serum uric acid levels was modest (0.8 mg/dL) and fully reversible after discontinuation of treatment. Further observations have shown that the incidence of gout is higher in patients with previous gout attack, especially if the baseline levels of uric acid exceed the upper limit of normality [19], but, in general, this should not be a concern in the daily clinical practice [20].

The incidence of decreased glomerular filtration rate (GFR) was 0.7% among patients treated with BA and 0.1% (*p *= 0.02) in the placebo arm [7]. BA increased the plasma levels of hepatic enzymes (2.8% versus 1.3%, *p *= 0.004) [7]. In all the cases, these elevations were asymptomatic, and aminotransferase levels returned to <3 ULN irrespective of whether the subjects continued or discontinued study treatment [20]. Despite BA has not been studied in patients with severe kidney and liver disease, including individuals with end-stage renal disease and Child-Pugh Class C hepatic impairment, the current opinion is that the pharmacokinetic changes in patients with renal and hepatic impairment are not clinically significant and are not expected to affect the efficacy or the safety profile of BA: therefore, no dosage adjustment is necessary [8, 20].

New-onset or worsening diabetes resulted significantly lower in BA treated patients (4.0% versus 5.6%; *p *= 0.03) [7], with an OR of 0.66 (95% CI 0.48 to 0.90), according to a meta-analysis that included 3629 patients [21].

A rare but potentially serious TEAE is the tendon rupture or injury which occurred in 10 (0.5%) out of 2009 BA patients compared to none of the 999 placebo individuals [19]. All of the tendon ruptures or injuries occurred in patients taking statins or with other risk factors such as fluoroquinolone or systemic corticosteroid use, diabetes, gout, rheumatoid arthritis, renal failure, age >60 years, male sex, and history of tendon disorders.

## 4. Clinical Practical Considerations

According to the characteristics described above, BA results in a very “easy-to-use” and “handy” molecule. Indeed, the single dose, once-daily, neutral to food intake administration, the theoretical absence of muscular effects (i.e., hepatic selectivity), the efficacy independent of the background therapy, the potentiality as add-on treatment, the favorable effect on hs-CRP and the not negative interaction with glucose metabolism give the physicians a wide spectrum of opportunities.

BA is available alone or in fixed-dose combination with ezetimibe. The choice between the two should be guided by the patient risk category, the percent of LDL-C reduction to achieve, and the background of other lipid lowering therapies. From a general point of view, taking BA and ezetimibe in a fixed dose combination than separately improves adherence and compliance and allows to modulate the statin types and doses reducing the risk of statin-associated muscle symptoms.

BA represents a valid new therapeutical option in controlling LDL-C in the elderly (>80 years). Although patients older than 80 years are underrepresented in the major trials and LDL-C target in the elderly remains controversial [22, 23], recent studies confirm the benefit of statins in reducing all cause and cardiovascular mortality in US veterans 75 years and older [24]. On the other hand, several national regulatory authorities do not allow the utilization of PCSK9i in patients aged 80 years and older. Still, the achievement of the LDL-C target should be accomplished also in the very old patients, especially if they are at very high/extreme risk. BA represents a strategic tool in such individuals considering that the CLEAR program did not exclude any participants because advancing age and, more important, the selectivity of the BA makes the molecule very suitable in the elderly, in term of tolerability and general safety. Nevertheless, the enrolled patients with age >80 years were a small subfraction and, overall, the mean age of the CLEAR studies population was about 65 ± 10 years. Moreover, caution must be taken when BA is utilized in elderly patients with hyperuricaemia or impaired renal function or on poly-pharmacotherapy.

As mentioned before, renal and hepatic impairment are of little or no concern with regard to BA utilization. Although no studies in end-stage renal disease and in patients with severe liver disease (Child-Pugh class C) are available, in most of the cases no dosage adjustment is necessary. Indeed, mean difference in creatinine levels at week 12 was 0.048 mg/dL for BA treatment versus –0.002 mg/dL for placebo [19]. These changes were observed within the first 4 weeks of treatment, were stable over time, and were reversible after treatment discontinuation. On the other hand, pooled data from RCTs have reported that treatment with BA was associated with slight elevations in the liver enzymes. The rate of repeated and confirmed (2 consecutive incidences) elevations in aminotransferase levels >3 times the upper limit of normal (ULN) was 0.8 per 100 person-years for BA subjects and 0.3 per 100 person-years for placebo. The incidence of aminotransferase elevations >5 × ULN was comparable between treatment groups being 0.3 per 100 person-years for subjects treated with BA and 0.2 per 100 person-years for placebo group, respectively [19]. In all cases these elevations were asymptomatic and aminotransferase levels returned to <3 the ULN irrespective of whether the subjects continued or discontinued study treatment, although the time for normalization is not reported in the literature [19].

A careful monitoring is required when BA is prescribed in individulas with a prior history of gout. In these cases a increase vigilance for hyperuricemia and gout is mandatory, but it should be not a reason to interrupt a priori the treatment, considering that (1) the clinical benefits of BA treatment might balance the potential risk of gout and (2) the fully reversibility of the elevation in acid uric after discontinuation of BA treatment. However, subjects with asymptomatic hyperuricemia (serum urate >6.8 mg/dL with no prior gout flares or subcutaneous tophi) do not require urate-lowering therapy [20].

In patients aged 60 and older, who are taking corticosteroids or fluoroquinolones, or with renal failure or history of tendon rupture, discontinuation of BA is mandatory if tendon rupture occurs. Withdrawal of BA is recommended in all patients with joint pain, swelling or inflammation [8]. Similarly, BA should be avoided in patients taking simvastatin >20 mg and pravastatin >40 mg, but not other statins and it not recommended in pregnant women and during the breastfeeding period.

## 5. Place in Therapy for the Cardiologists (Secondary Prevention)

**Fig. 1.**
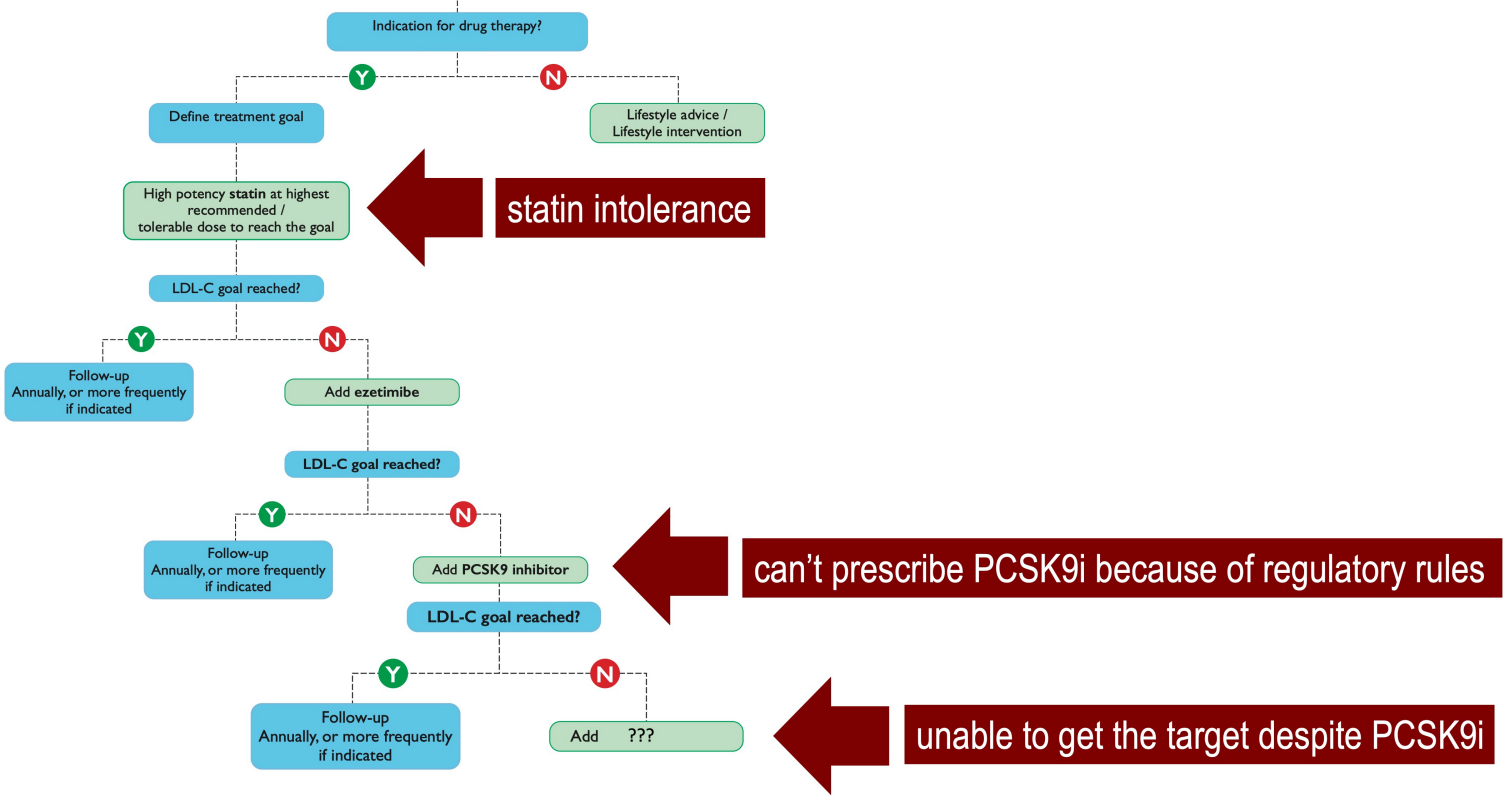
**The stepwise therapeutical strategy according to the recommendations of the 2019 ESC/EAS guidelines for the management of dyslipidemias [1], in which single statin, dual statin/ezetimibe and triple statin/ezetimibe/PCSK9 inhibitor therapies are gradually introduced every 4 weeks until the achievement of LDL-C target**.

In patients with high/very high cardiovascular risk, current European guidelines for the management of dyslipidemias recommend a stepwise strategy starting with maximum tolerated dose of high intensity statin, followed by a combination with ezetimibe if the goals are not achieved after 4 weeks. If the further subsequent 4-week treatment of statin/ezetimibe dual therapy fails to reach the target, the addition of PCSK9 inhibitor is then recommended [1]. From a theoretical point of view, this step-by-step approach in lipid lowering treatment should guarantee the achievement of the therapeutical goals in all the patients, considering that the triple therapy (statin/ezetimibe/PCSK9i) is able to reduce the baseline LDL-C levels by 85%. This strategy, however, is affected by, at least, three potential barriers (Fig. 1, Ref. [1]), without considering other practical obstacles such as the cost and/or the route of administration.

First, the high proportion of patients with intolerance to statins, and, in particular, statin-associated muscle symptoms who likely contribute significantly to the very high discontinuation rates of statin therapy (up to 75%) within 2 years of initiation [25]. Second, when the patients are not eligible for PCSK9i because national regulatory agencies do not allow to prescribe them and/or do not recognize their reimbursement, if specific criteria are not fully satisfied. As result, it has recently been reported that, in Europe, patients initiated on PCSK9i had baseline LDL-C levels almost 3 times higher than the reccomended threshold for PCSK9i use [26]. Third, when despite the use of a multiple lipid lowering therapy (i.e., triple therapy: statin/ezetimibe/PCSK9i), the patient is unable to reach the recommended goal, especially if the subject is at very high (LDL-C should be <55 mg/dL) or extreme (LDL-C <40 mg/dL) ASCVD risk. In all these cases BA, as replacement (statin intolerance) or as add-on therapy may represent an additional choice to optimize the treatment and to guarantee the target or, at least, the proximity to it.

BA is not a competitor of PCSK9i, but in some circumstances it might be used before PCSK9i, being more cost-effective compared to more expensive therapies. In particular, in high and very high ASCVD risk patients not at LDL-C goal, BA alone might be utilized, on top of high intensity statin plus ezetimibe, when the distance from LDL-C goal is less than 20%. BA in FDC with ezetimibe, instead, might be used, on top of high intensity statins, when the distance from LDL-C goal is less than 40%.

Finally it should be recognize that, although clinical studies enrolled mainly patients at high and very high risk, BA or BA/ezetimibe in FDC might be used in moderate ASCVD risk patients, to contribute to the LDL-C reduction and might have a role in primary prevention, especially in patients with insulin resistance, metabolic syndrome and non-alcoholic fatty liver disease.

**Fig. 2.**
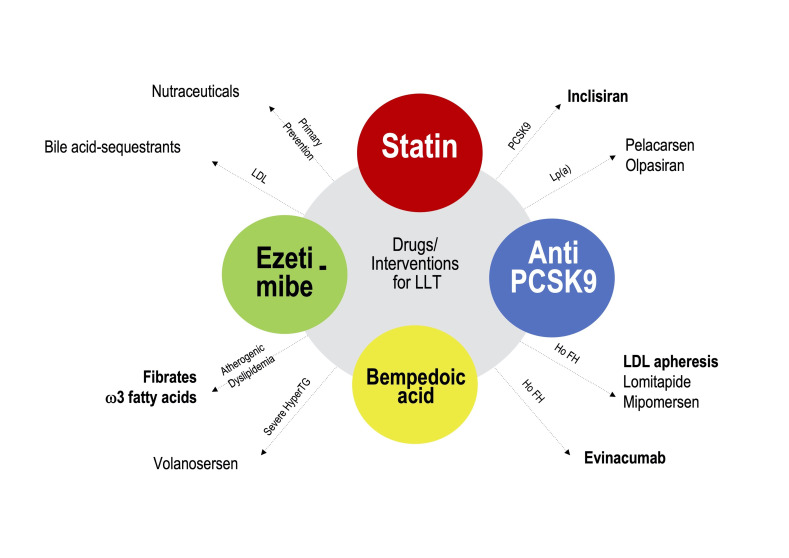
**The current lipid lowering available drugs**. Modified from: Johann Bauersachs, Heart failure drug treatment: the fantastic four, Eur Heart J (2021) 42, 681–683. (Ref. [27])

In conclusion, the pharmacokinetic and pharmacodynamic characteristics of BA make this drug a very useful tool in control LDL-C especially in high/very high/extreme ASCVD risk patients which, in a framework of personalized medicine, will play, along with other lipid lowering therapies, an important role in Preventive Cardiology. Indeed, in the next years, several molecules are expected to be introduced in the clinical practice. Many of them will permit an individual tailoring of the therapies directed non only toward LDL-C control but also to non LDL-C targets such as anti-apoCIII or anti-ANGPTL3 agents or anti-Lp (a) RNA therapeutics. For now, however, as illustrated in Fig. 2 (Ref. [27]), BA rapresents along with statin, ezetimibe and PCSK9i one of the 4 pillars of the modern lipid lowering treatment.

## Abbreviations

ASCVD, atherosclerotic cardiovascular diseas; BA, Bempedoic acid; CV, cardiovascular; CAD, Coronary Artery Disease; FDC, fixed-dose combination; GFR, glomerular filtration rate; HeFH, heterozygous familial hypercholesterolemia; hs-CRP, high sensitive C-reactive protein; HMGCR, Hydroxy-methylglutaryl coenzyme A reductase; LDL-C, LDL-cholesterol; TEAE, Treatment-emergent adverse events; ULN, upper limit of normal; ACSVL1, Very-long-chain acyl-CoA synthetase-1.
